# Mental Disorders in Megacities: Findings from the São Paulo Megacity Mental Health Survey, Brazil

**DOI:** 10.1371/journal.pone.0031879

**Published:** 2012-02-14

**Authors:** Laura Helena Andrade, Yuan-Pang Wang, Solange Andreoni, Camila Magalhães Silveira, Clovis Alexandrino-Silva, Erica Rosanna Siu, Raphael Nishimura, James C. Anthony, Wagner Farid Gattaz, Ronald C. Kessler, Maria Carmen Viana

**Affiliations:** 1 Section of Psychiatric Epidemiology–LIM 23, Department and Institute of Psychiatry, University of São Paulo Medical School, São Paulo, Brazil; 2 Department of Preventive Medicine, Federal University of São Paulo, São Paulo, Brazil; 3 Institute of Social Research, University of Michigan, Ann Arbor, Michigan, United States of America; 4 Department of Epidemiology, College of Human Medicine, Michigan State University, East Lansing, Michigan, United States of America; 5 Laboratory of Neuroscience–LIM 27, Department and Institute of Psychiatry, University of São Paulo Medical School, São Paulo, Brazil; 6 Department of Health Care Policy, Harvard Medical School, Boston, Massachusetts, United States of America; Wayne State University, United States of America

## Abstract

**Background:**

World population growth is projected to be concentrated in megacities, with increases in social inequality and urbanization-associated stress. São Paulo Metropolitan Area (SPMA) provides a forewarning of the burden of mental disorders in urban settings in developing world. The aim of this study is to estimate prevalence, severity, and treatment of recently active DSM-IV mental disorders. We examined socio-demographic correlates, aspects of urban living such as internal migration, exposure to violence, and neighborhood-level social deprivation with 12-month mental disorders.

**Methods and Results:**

A representative cross-sectional household sample of 5,037 adults was interviewed face-to-face using the WHO Composite International Diagnostic Interview (CIDI), to generate diagnoses of DSM-IV mental disorders within 12 months of interview, disorder severity, and treatment. Administrative data on neighborhood social deprivation were gathered. Multiple logistic regression was used to evaluate individual and contextual correlates of disorders, severity, and treatment. Around thirty percent of respondents reported a 12-month disorder, with an even distribution across severity levels. Anxiety disorders were the most common disorders (affecting 19.9%), followed by mood (11%), impulse-control (4.3%), and substance use (3.6%) disorders. Exposure to crime was associated with all four types of disorder. Migrants had low prevalence of all four types compared to stable residents. High urbanicity was associated with impulse-control disorders and high social deprivation with substance use disorders. Vulnerable subgroups were observed: women and migrant men living in most deprived areas. Only one-third of serious cases had received treatment in the previous year.

**Discussion:**

Adults living in São Paulo megacity had prevalence of mental disorders at greater levels than similar surveys conducted in other areas of the world. Integration of mental health promotion and care into the rapidly expanding Brazilian primary health system should be strengthened. This strategy might become a model for poorly resourced and highly populated developing countries.

## Introduction

World population growth over the next quarter-century is projected to be heavily concentrated in urban areas, especially in megacities of the developing world, with area population greater than 10 million. Associated trends may include increases in social and economic inequalities, stressors linked to rapid urbanization, and related deterioration in health, particularly mental illnesses [1]. This process has already started in a number of sentinel areas that can be studied to provide a forewarning of the future of health in developing countries. Although its unique historical, economic, and cultural backgrounds distinguish the São Paulo Metropolitan Area (SPMA) from other megacities in the developing world, it could be viewed as one such area and it was chosen to conduct the São Paulo Megacity Mental Health Survey (SPMHS), the Brazilian segment of the World Mental Health (WMH) Survey Initiative, under the auspices of the World Health Organization [2].

Located in southeastern Brazil, SPMA holds more than 10% of the Brazilian population [3] and is the fifth largest metropolitan area in the world, with around 20 million inhabitants. It is regarded as an especially important industrial and commercial center in the Latin America and Caribbean (LAC) region. Between 1997 and 2007, the urbanization process increased the population by 16%: 10% in the city of São Paulo and 25% in peripheral areas and surrounding municipalities [3]. This growth is partially a consequence of rural-to-urban mobility of migrants from the poor regions of Brazil to the outskirts of SPMA, who seek job opportunities, education, medical care, and better living conditions [4]. As in other metropolitan areas [1], these changes lead to inordinate land occupation, housing shortage, widespread of informal work sector, and aggravating social deprivation in some neighborhoods [5], [6]. This environmental context also increases the level of social isolation and dissolution of primary family relations. Associated impoverishment can yield escalated violence and homicide rates, with resulting dissemination of insecurity over the metropolitan area [7], [8]. All these structural and psychosocial circumstances mirror and underscore historical social inequalities and long-term income disparities in Brazil [9].

The impact of living in urban areas at a given moment in time [5], or exposure to urbanicity [1], along with individual factors may have consequences for mental health [10], [11], [12], [13]. Knowledge on how urbanicity can affect mental health is still limited, but has been described as a priority [13], [14].

With respect to Brazil, the 2005 estimates of the Global Burden of Disease Project [15] suggest that neuropsychiatric conditions accounting for 21.5% of all disability-adjusted life years (DALYs) (25.1% in women and 18.6% in men). Nevertheless, most of the data for these estimates have come from limited psychiatric epidemiologic studies [16] carried out in small communities [17], or selected neighborhoods in large cities [18], [19], [20], without providing comprehensive information about severity and disability.

The current report aims extending previous Brazilian psychiatric surveys with data on prevalence and severity levels of recently active DSM-IV mental disorders. The use of services was assessed to guide planning and implementation of health services policies [21], [22], [23]. Also, along with examination of socio-demographic correlates, we inspected, in a general framing, some characteristics of urban life (i.e., migration status, exposure to crime-related traumatic events, exposure to an urban environment, and neighborhood social deprivation level) in relation to active mental disorders.

## Methods

### Ethics Statements

The SPMHS procedures for recruitment, obtaining informed consent, and protecting human subjects during field procedures were approved by the Research and Ethics Committee of the University of São Paulo Medical School. Respondents were interviewed only after informed written consent was obtained, and total confidentiality was assured. Eligible respondents were those who were 18 or older, Portuguese-speaking, and without any disability or handicap that would otherwise impair their ability to participate in the interview.

### Sample

The SPMHS was designed to be a representative sample survey of household residents aged 18 years and older in the SPMA, an area formed by the state capital city of São Paulo and its 38 surrounding municipalities, covering a geographical area of 8,051 km^2^ [24]. At the time of data collection (May 2005 to May 2007), 11 million inhabitants were 18 years or older [24].

Detailed descriptions of sampling and weighting methods are presented elsewhere [2]. Briefly, respondents were selected through a stratified, multistage area probability sample of households. Within each household one respondent per dwelling was selected through a Kish table. In all strata, the primary sampling units (PSUs) were 2,000 cartographically defined census count areas [24]. Each municipality contributed to the total sample size according to its population size.

The sample size, after sampling, recruitment, and informed consent, was 5,037. Initially, 7,700 households were selected to achieve the planed sample of 5,000 subjects, allowing for a 35% non-response rate. Using the strategy of releasing consecutive sub-samples of 500 households, representing a random sub-sample of the whole sampling frame, the fieldwork was therefore interrupted with the release of the first 6,199 housing units selected. A total of 5,237 subjects agreed to participate, but 200 elderly respondents were considered not eligible due to cognitive impairment. The overall survey participation level was 81.3%.

### Measures

#### Diagnostic Assessment

Respondents were assessed using the Composite International Diagnostic Interview (WMH-CIDI), a fully structured lay interview that generates diagnoses according to the DSM-IV criteria, translated and adapted to the Brazilian-Portuguese language. Face-to-face interviews were carried out by professional interviewers who received five-day standardized training.

The Brazilian version of this instrument consisted of two parts. Part 1 included core diagnostic sections (major depression, mania, panic disorder, specific phobia, social phobia, agoraphobia, generalized anxiety disorders [GAD], adult separation anxiety [ASA], substance use disorders [SUD], intermittent explosive disorder [IED], attention-deficit/hyperactivity disorder [ADHD], oppositional-defiant disorder [ODD], and conduct disorder), and suicidal behavior. Additional sections with demographic information, daily functioning, and physical morbidity were administered to all respondents (n = 5,037). Part 2 included questions about risk factors, consequences, and other correlates, along with assessments of additional disorders (posttraumatic stress disorder [PTSD], obsessive-compulsive disorder [OCD]), and were administered to all respondents who met lifetime criteria for any disorder in Part 1, plus a probability subsample of other respondents in an effort to reduce the respondent's burden and control the costs of the study. Part 2 was administered to 2,942 respondents. DSM-IV disorders active within 12 months prior to the date of assessment are considered herein to include four classes of disorders as follows: anxiety disorders (panic disorder, GAD, agoraphobia without panic disorder, specific phobia, social phobia, PTSD, OCD, ASA), mood disorders (major depressive disorder, dysthymia, bipolar disorder I or II), impulse-control disorders [ICD] (ODD, conduct disorder, ADHD, IED), and SUD (alcohol and drug abuse and dependence). DSM-IV organic exclusion rules were used in making the diagnoses. Hierarchy rules were also used in diagnosing major depressive disorder, dysthymia, GAD, and ODD. For SUD, DSM-IV abuse was defined with or without dependence in recognition abuse often being a stage in the progression to dependence. Hierarchy-free diagnoses were consistently used in analyses of comorbidity.

Blind clinical re-interviews using the Structured Clinical Interview for DSM-IV Axis I disorder (SCID-I) [25] with a probability subsample of WMH respondents found generally good agreement between WMH-CIDI diagnoses and SCID diagnoses [26]. Preliminary results of the clinical reappraisal study in the SPMHS with a probability subsample of 775 respondents (not included in the previous validation study cited above) showed a good total classification accuracy (range:76%–99%) and an area under the Receiver Operating Characteristics curve around 0.7 for any disorder.

#### Severity

In order to give information on clinical significance and needs assessment, the WMH-CIDI includes extensive information on symptom persistence, distress, and associated disability, allowing the classification of cases in severity levels based not only in psychopathology or symptoms, but also taking into account impairment in several domains of functioning. Each diagnostic section contains explicit questions about impairment in various areas of functioning among 12-month active cases. Four of these questions are the Sheehan Disability Scale (SDS), which ask respondents to rate the impairment caused by a focal disorder during one month in the past year when it was more severe. The SDS assessed disability in work role performance, household maintenance, social life, and intimate relationship on 0–10 visual analog scales with verbal descriptors and associated scale scores of none, 0; mild, 1–3; moderate, 4–6; severe 7–9, and very severe, 10 [27].

Active cases were classified as ‘severe’ if they had any of the following: (1) bipolar I disorder; (2) substance dependence with physiologic signs; (3) ever attempted suicide in the last 12 months and had at least one 12-month disorder; and (4) more than one 12-month disorder and a high level of impairment on Sheehan Disability Scales i.e. at least 3 out of the 4 of the following must be true: score≥8 in household maintenance domain, ≥7 in work role performance domain, ≥8 in intimate relationship domains, and ≥7 in social life domain. Among those who are not categorized as severe cases, respondents are labeled ‘moderate’ if they had at least one disorder with a moderate level of impairment on any SDS domain or substance dependence without physiological signs. The remaining respondents with any active disorder were categorized as ‘mild’.

#### Service Use

Treatment was assessed by asking respondents if they ever saw any professional for problems with their emotions, nerves, mental health, or use of substances [28]. Twelve-month treatment variables were created using a combination of disorder-specific treatment questions and details about services received from particular providers. Broad categories of health care and non-health care providers were created. Health care providers included mental health care professionals (psychiatrist, psychologist, social worker, mental health counselor) and general medical providers seen for treatment of emotional problems (primary care physician, other general physician, nurse, or any other health care professional). Non-health care providers included human services professionals (religious or spiritual advisor; counselor in a non-mental setting; complementary-alternative medicine [CAM] provider, as a chiropractor or folk healer; and self-help group).

### Correlates

#### Socio-demographic

Socio-demographic correlates included age (18–34, 35–49, 50–64, and 65+ years old), gender (male/female), completed years of education (0–4, 5–8, 9–11, and ≥12; referred to as low, low-average, high-average, and high, respectively), marital status (married/cohabiting, previously married, never married), and family income. Family income was defined in categories based on the respondent's household income per family member divided by the median income-per-family-member in the entire sample. Household income was defined as low if this ratio was 0.5 or less, low-average if the ratio was in the range 0.5–1.0, high-average if 1.0–2.0, and high if greater than 2.0.

#### Exposure to crime-related traumatic events

Considering previous evidence of endemic urban violence in the SPMA [7] and the adverse consequence of crime victimization on mental health [29], seven crime-related traumatic events were selected from the list of events from the PTSD section of CIDI: i. kidnapped or held captive, ii. ‘quicknapping’ (a short term kidnapping), iii. stalked, iv. mugged or threatened with a weapon, v. witnessed anyone being injured or killed, or unexpectedly saw dead body, vi. witnessed atrocities or carnage, vii. witnessed a close person to be kidnapped, tortured or raped. The exposure to crime-related traumatic events was summarized as: none, one event, two events, and three or more events.

#### Exposure over early years of the life course to urban environment (urbanicity)

Respondents were asked if they were raised (i.e., spent most of their childhood and adolescence) in a large city or its suburbs, a small town or village, or a rural area. To address the impact of exposure over early years of the life course to urban environment as correlates of the four classes of disorders and disorders severity considered herein, three dummy variables were created to reflect level of exposure to urbanicity [5]. Those reported being raised in rural areas were considered with the lower exposure to urbanicity, followed by those raised in small town or village (medium level of exposure). Those raised in large cities were considered the highest level of exposure to urbanicity.

#### Migration status

To assign the migrant status, respondents were asked if were born outside SPMA and their age of migration.

#### Neighborhood social deprivation level

An index of neighborhood social deprivation (NSD) level was developed by the Center of Metropolitan Studies (http://www.centrodametropole.org.br) [6] and assigned to each census unit, to reflect social conditions in the SPMA geographical space using data from the 2000 Census [24]. This index represents a combination of socio-economic deprivation dimension (income, level of education, family size, and percentage of families headed by a woman with low educational level) and the population's age structure. The NSD index ranges from 1 (no social deprivation) to 8 (high social deprivation; see Table S1 for details), with a concentric spatial distribution of deprivation increasing in peripheral neighborhoods. These eight levels were summarized in three indicators: no/low (combined index of 1, 2, and 3 NSD level), medium-low/medium (6 and 4), and high/very high NSD (5, 7, and 8).

### Analytic approach

In order to consider the stratified multi-stage sample design, the analytic approach included conventional methods for variance estimation with complex sample survey data, and weights were used to adjust for differences in within-household probability of selection and non-response. A post-stratification weight was used to make the sample distribution comparable to the population distribution in the year 2000 Census on a cross-classification of socio-demographic variables (see [2] for details). Weights were used to address the coverage of survey variables in Part 1 and Part 2 of the assessment, with an additional weight used when Part 2 variables are considered (e.g., urbanicity; migration status, crime-related traumatic events).

Prevalence estimates within sub-samples were obtained with cross-tabulations. Multiple logistic regression analysis was used to study correlates of prevalence, disorder severity (severe/moderate vs. mild), and treatment. Analyses of correlates were conducted in three stages. First, multiple logistic regression models were built to examine the association between outcomes and socio-demographic characteristics. A second set of models was created to examine the exposure to crime-related traumatic events controlling for gender, age, and NSD, as correlates of the four classes of disorders. Third, models were elaborated to consider the potentially separable associations with migration status, level of exposure to urbanicity, and NSD, as correlates of the four classes of disorders and disorder severity, controlling for socio-demographic factors (gender, age, income, marital status, and education). These analyses were then repeated with the addition of higher order product-terms between gender (given its important modifier effect), migration status, level of exposure to urbanicity, and NSD to study whether the association of each of these factors was uniform across each class of disorder and disorder severity. When necessary, a stepwise backward approach was used to select between intercorrelated variables.

Logistic regression coefficients and standard errors (SEs) were exponentiated. The resulting estimates are reported here as odds-ratios (OR) with 95% confidence intervals (CIs). As the survey data used in the analysis were weighted and the sample was geographically clustered, SEs were based on the design-based Taylor series linearization method. These calculations were made using SUDAAN software [30]. Wald χ^2^ tests calculated from Taylor series coefficient variance-covariance matrices were used to evaluate the statistical significance of sets of coefficients, with two-sided alpha set at 0.05.

## Results

### Prevalence and severity

The proportion of SPMA household residents with at least one recently active DSM-IV/CIDI disorder under study was 29.6%, and these cases were evenly distributed across the severity gradient from mild (33.2%), moderate (33.0%) to severe (33.9%), such that about one in ten residents had a recently active severe mental disorder (10%). Anxiety (19.9%) and mood disorders (11%) were the most prevalent classes of disorder, followed by disturbances of impulse-control (4.2%), and substance use disorders (3.6%). Table 1 shows these estimates along with specific disorders. For example, 9.4% respondents suffered from recently active major depressive disorder and about one in nine residents (10.6%) had recent specific phobia.

**Table 1:** Estimated Twelve-Month Prevalence and Severity of DSM-IV/WMH-CIDI disorders: results from the São Paulo Megacity Mental Health Survey (SPMHS).

					Severity^a^					
			12-Month Prevalence		Mild		Moderate		Serious	
Disorder Category	Disorder	n	%	(SE)	%	(SE)	%	(SE)	%	(SE)
**Anxiety Disorders**	Panic disorder^b^	61	1.1	0.2	15.7	6.7	27.7	6.9	56.6	6.8
	Generalized anxiety disorder^b^	134	2.3	0.2	21.3	4.7	36.8	5.8	41.9	4.7
	Social phobia^b^	186	3.9	0.3	10.6	2.3	33.7	4.8	55.6	6.2
	Specific phobia^b^	572	10.6	0.5	33.9	3.2	31.0	3.7	35.0	2.4
	Agoraphobia without panic^b^	88	1.6	0.3	23.9	5.7	18.6	5.5	57.4	5.8
	Post-traumatic stress disorder^c^	81	1.6	0.2	18.3	5.9	30.6	5.5	51.1	7.2
	Obsessive-compulsive disorder^c^	155	3.9	0.4	30.0	4.0	27.4	4.0	42.5	4.5
	Adult separation anxiety disorder^b^	111	2.0	0.3	15.4	4.4	33.4	4.6	51.1	5.4
	**Any anxiety disorder** c	841	19.9	0.8	31.7	2.2	31.8	2.4	36.5	2.2
**Mood Disorders**	Dysthymia^b^	62	1.3	0.3	13.3	4.8	35.8	8.0	50.9	8.6
	Major depressive disorder^b^	491	9.4	0.6	18.0	1.9	38.9	2.7	43.1	3.5
	Bipolar I and II disorders^b^	73	1.5	0.2	6.1	3.2	28.5	8.4	65.4	8.5
	**Any mood disorder** b	570	11.0	0.6	16.4	1.6	37.6	2.4	46.0	3.3
**Impulse-control Disorders**	Oppositional-defiant disorder^b^	22	0.5	0.2	26.3	11.7	24.0	9.8	49.7	12.2
	Conduct disorder^b^	19	0.5	0.1	1.5	1.5	16.5	10.7	81.9	10.8
	Attention deficit disorder^b^	49	0.9	0.2	25.2	8.1	11.6	4.9	63.2	8.8
	Intermittent explosive disorder^b^	138	3.1	0.3	36.6	6.8	27.9	5.2	35.5	5.6
	**Any impulse-control disorder** b	199	4.2	0.4	34.0	5.7	26.6	4.3	39.3	4.4
**Substance Use Disorders**	Alcohol abuse^b^	135	2.7	0.3	36.5	4.9	16.3	3.6	47.2	4.0
	Alcohol dependence^b^	64	1.3	0.2	0.0	0.0	5.5	2.0	94.5	2.0
	Drug abuse^b^	31	0.6	0.1	11.7	3.4	14.7	9.0	73.7	8.9
	Drug dependence^b^	21	0.5	0.1	0.0	0.0	6.8	6.5	93.2	6.5
	**Any substance use disorder** b	164	3.6	0.4	28.2	4.1	15.5	3.6	56.3	3.8
**Any 12-month Disorder**	Any^c^	1277	29.6	1.0	33.1	1.4	33.0	1.8	33.9	1.4
	0 Disorders^c^	1665	70.4	1.0						
	1 Disorder^c^	733	17.8	0.7	46.1	2.0	34.4	2.4	19.5	1.8
	2 Disorders^c^	264	5.9	0.4	21.7	3.2	38.1	4.2	40.2	3.3
	3+ Disorders^c^	280	5.8	0.5	5.4	1.9	23.3	3.2	71.3	3.4
**Severity**	Serious^c^	468	10.0	0.6						
	Moderate^c^	412	9.8	0.5						
	Mild^c^	397	9.8	0.6						

Percentages in the three severity columns are repeated as proportions of all cases and sum to 100% cross each row.

Part 1 Total Sample Size = 5037, Part 2 Total Sample Size = 2942.

a Severity calculated using Part 1 weights.

b Part1 sample, prevalence calculated using Part 1 weights.

c Part2 sample, prevalence calculated using Part 2 weights.

While most cases had just one active mental disorder, there was some comorbidity: about one in 16 respondents were found to have two active disorders (5.9%) and a roughly equal number have three or more disorders (5.8%). Severity was strongly related to comorbidity: an estimated one in five of the cases (19.5%) with only one disorder qualified as ‘severe’. By comparison, the corresponding severity estimates were 40.2% for comorbid cases with two disorders, and 71.3% for those with more than two active disorders.

The distribution of severity also varied across classes of disorders, with the highest percentage of serious cases for SUD (56.3%) and the lowest for anxiety disorders (36.5%). Nonetheless, a majority of cases of certain anxiety disorders also qualified as ‘severe’: agoraphobia without panic (57.4%), panic disorder (56.6%), and social phobia (55.6%). Among mood disorders, bipolar disorder had the highest percentage of ‘severe’ cases (65.4%), which include all active cases of bipolar I disorders and cases of bipolar II associated with suicide attempt in the last 12-month, or associated with high impairment in Sheehan Disability Scale. Among ICD, 82% of conduct disorders were classified as ‘severe’. The highest percentages of severe cases were found for alcohol and drug dependence (94.5% and 93.2%, respectively).

Table S2 shows the prevalence of 12-month disorders by gender and age cohorts. Anxiety disorders were more frequent in women, but no gender differences were observed in social phobia, obsessive-compulsive disorders, and adult separation anxiety. Considering mood disorders, major depression and dysthymia were more frequent in women, whereas there was no gender differences for bipolar I or II. Only two of the four impulse control disorders assessed were more frequent in males: conduct and attention deficit disorders. Oppositional-defiant disorder and intermittent explosive disorders were equally distributed across gender. In three of the SUD, males had prevalence four times higher than females, with the exception of drug dependence.

There are significant inter-cohorts variations for the four broad classes of disorders, with prevalence declining in the eldest cohort, with the exception of GAD, agoraphobia without panic, and dysthymia, in which no inter-cohort variation occurred.

### Socio-demographic correlates of disorders and severity

Table 2 shows the results of modeling the occurrence of recently active case status, and severity level, as a function of socio-demographic variables. Based on these estimates, women were more likely to have mood, anxiety, and severe/moderate disorders than men, while men were more likely to have SUD than women (all p<0.05). For the ICD, no male-female differences were found. The three younger age groups were more likely than the oldest age group to qualify as recently active cases of mood, anxiety, SUD, and severe/moderate disorders. For ICD, active case status was concentrated in early adulthood. Being previously married was associated with an increased likelihood of presenting mood, anxiety, ICD, and severe/moderate disorders. Being in the lower income strata was associated with a reduced likelihood of ICD. Respondents with less than some primary education were more likely to present anxiety disorders, and those with less than college education being more likely to present SUD.

**Table 2:** Socio-demographic correlates of summary categories of 12-month DSM-IV/WMH-CIDI disorders, and severity.

Variable		Any mood disorder OR (95% CI)	Any anxiety disorder OR (95% CI)	Any impulse disorder OR (95% CI)	Any substance disorder OR (95% CI)	Any severe/moderate 12-month disorder OR (95% CI)
**Gender**	Male	1.0	1.0	1.0	1.0	1.0
	Female	2.7 (1.8–3.8)	2.2 (1.6–3.1)	0.9 (0.6–1.3)	0.2 (0.2–0.4)	1.9 (1.5–2.5)
	Wald *χ* ^2^	31.7^a^	28.9^a^	0.4	41.5^a^	28.7^a^
**Age, years**	18–34	4.2 (2.2–8.3)	3.0 (1.9–4.8)	8.7 (1.5–51.9)	22.0 (4.4–109.8)	4.4 (2.2–8.8)
	35–49	4.9 (2.7–8.9)	2.9 (1.8–4.8)	4.5(0.8–25.3)	17.8 (3.6–88.0)	3.9 (1.8–8.1)
	50–64	3.5 (2.0–6.2)	3.4 (1.7–7.0)	2.6 (0.3–24.1)	7.7 (1.6–37.0)	3.5 (1.9–6.6)
	65 ^+^	1.0	1.0	1.0	1.0	1.0
	Wald *χ* ^2^	31.6^a^	24.0^a^	16.9^a^	24.8^a^	18.8^a^
**Income**	Low	1.1 (0.6–2.0)	0.9 (0.6–1.4)	0.4 (0.2–0.9)	0.6 (0.3–1.3)	1.0 (0.6–1.5)
	Low-Average	1.5 (0.8–2.8)	1.2 (0.8–1.7)	0.4 (0.2–1.0)	0.6 (0.3–1.3)	0.9 (0.6–1.5)
	High-Average	1.1 (0.6–1.8)	1.2 (0.8–1.7)	0.4 (0.2–0.8)	0.8 (0.3–1.6)	1.1 (0.7–1.6)
	High	1.0	1.0	1.0	1.0	1.0
	Wald *χ* ^2^	3.9	5.9	8.5^a^	1.8	2.3
**Marital Status**	Married/Cohabiting	1.0	1.0	1.0	1.0	1.0
	Sep/Widowed/Div	1.7 (1.3–2.3)	1.5 (1.1–2.0)	2.0 (1.2–3.3)	1.3 (0.7–2.5)	1.7 (1.3–2.2)
	Never Married	1.0 (0.7–1.5)	1.1 (0.8–1.5)	0.9 (0.5–1.7)	1.8 (0.9–3.5)	1.3 (0.9–1.7)
	Wald *χ* ^2^	15.2^b^	7.2^a^	9.0^a^	3.4	16.4^a^
**Education**	Low	0.9 (0.5–1.5)	1.5 (1.0–2.4)	1.7 (0.7–4.4)	4.8 (2.2–10.7)	1.3 (0.8–2.0)
	Low-Average	1.1 (0.8–1.7)	1.4 (0.8–2.4)	2.2 (1.0–4.8)	3.5 (1.3–9.6)	1.4 (0.9–2.3)
	High-Average	1.1 (0.8–1.5)	1.0 (0.6–1.6)	1.7 (0.9–3.1)	2.1 (0.9–4.6)	1.2 (0.9–1.7)
	High	1.0	1.0	1.0	1.0	1.0
	Wald *χ* ^2^	1.7	14.2^a^	5.4	17.4^a^	2.2

a p<0.05, two-sided test.

### Crime-related traumatic events

The experience of crime-related traumatic events was common in the sample. An estimated 54.6% (SE:1.5) of the area residents had experienced at least one of these events at some time in their life. An estimated 6.1% (SE:0.5) of respondents had experienced three or more crime-related events, and 17.6% (SE:1.0) had experienced two events.

Among the seven crime-related traumatic events examined, six were associated to at least one disorder (p<0.05). These events were: witnessed anyone being injured or killed, or unexpectedly saw dead body (experienced by 35.7% of respondents), being mugged or threatened with a weapon (34%), being stalked (5.5%), seeing a close person being kidnapped, tortured or raped (5.2%), witnessing atrocities or carnage (3.5%), being kidnapped or held captive (0.5%). ‘Quicknapping’ (experienced by 1.6%) was not associated with any disorder. Being a recently active mental disorder case was associated with the number of traumatic events experienced (Table 3). Elevated odds for mood, anxiety, and ICD were observed even for those who had experienced only one of the events considered. An exception was SUD, where an increased odd was found only among residents who had experienced three or more of these traumatic events.

**Table 3:** Associations of exposure to crime-related§ traumatic events with 12-month DSM-IV/WMH-CIDI disorders and disorder severity.

Number of events	n	Any mood disorder OR (95% CI)†	Any anxiety disorder OR (95% CI)	Any impulse disorder OR (95% CI)	Any substance disorder OR (95% CI)	Severe % (SE) ††	Moderate % (SE)	Mild % (SE)	No disorder % (SE)
**3 or more**	222	3.8 (2.3–6.1)	3.5 (2.3–5.3)	5.2 (2.4–11.4)	5.7 (3.2–10.2)	23.8 (3.0)	16.8 (2.8)	16.8 (2.7)	42.6 (4.3)
**2**	563	2.1 (1.6–2.9)	1.7 (1.3–2.4)	2.3 (1.3–4.0)	1.6 (0.9–2.7)	12.4 (1.2)	10.9 (1.5)	7.6 (1.4)	69.1 (2.1)
**1**	966	1.6 (1.2–2.0)	1.6 (1.2–2.1)	2.1 (1.4–3.1)	1.0 (0.6–1.6)	10.5 (0.8)*	9.5 (1.2)*	10.7 (1.1)	69.3 (1.5)*
**0**	1191	1.0	1.0	1.0	1.0	6.9 (0.8)*	8.5 (0.7)*	9.1 (0.9)	75.5 (1.5)*
**Wald ** ***χ*** **^2^_3_; p**		36.63; <0.0001	39.09; <0.0001	23.85; <0.0001	38.22; <0.0001				

Data from the Part 2 sample (n = 2,942).

§ Kidnapped, threatened with a weapon, stalked, other tortured, other killed, atrocities, quicknapped.

† Based on multivariate logistic regression analysis controlling for gender, age, and neighborhood social deprivation.

†† Standard Error.

* Significantly different from the prevalence in the 2 or 3 or more sub-samples at .05 level, two-sided test.

Exposure to urban violence was also associated with the severity distribution of these disorders. About 40% of those exposed to three or more events met criteria for a severe/moderate disorder, declining to around 20% for those exposed to one or two trauma.

### Exposure over early years of the life course to urban environment

Roughly three of five SPMA urban residents were raised in an urban area, spending most of their childhood and adolescent years in SPMA or some other large urban center (57.9%; SE:1.8). About one in four had been raised in a small city (23.6%; SE:1.3), and only one in 5–6 were raised in rural areas (18.5%; SE:1.3). There was an excess occurrence of ICD in individuals raised in an urban area versus those raised in a rural area, as signified by an OR estimate of 1.8 (p<0.05; Table 4). Other mental disorders were not associated with this urbanicity variable.

**Table 4:** Associations (odds-ratio) of lifetime exposure of urbanicity, neighborhood social deprivation level, and migration status with 12-month DSM-IV/WMH-CIDI disorders.

	Any Mood Disorder OR (95% CI)†	Any Anxiety Disorder OR (95% CI)†	Any Impulse Disorder OR (95% CI)†	Any Substance Use Disorder OR (95% CI)†	Severity OR (95% CI)†
**Lifetime exposure of urbanicity**					
Rural areas	1.0	1.0	1.0	1.0	1.0
Small town	1.3 (1.0–1.8)	1.1 (0.8–1.7)	1.9 (1.1–3.3)	1.2 (0.6–2.2)	1.2 (0.9–1.6)
Large cities	1.4 (1.0–2.1)	1.0 (0.7–1.5)	1.8 (1.0–3.4)	1.2 (0.5–2.5)	1.3 (1.0–1.8)
Wald χ^2^/p	ns	NS	6.03/0.049	NS	NS
**Neighborhood Social Deprivation level** *					
No-low NSD	1.0	1.0	1.0	1.0	1.0
Low-medium/medium NSD	0.9 (0.7–1.1)	1.1 (0.9–1.3)	0.9 (0.6–1.5)	1.2 (0.7–2.1)	1.3 (1.0–1.6)*
High/very high NSD	0.8 (0.6–1.1)	0.9 (0.7–1.2)	1.3 (0.7–2.3)	1.8 (1.1–3.0)	1.3 (1.0–1.7)
Wald χ^2^/p	NS	NS	NS	5.8/0.02	NS
**Migration status**					
Non-migrants	1.0	1.0	1.0	1.0	1.0
Migrants	0.8 (0.6–0.9)	1.0 (0.8–1.3)	0.7 (0.4–1.1)	0.7 (0.4–1.3)	0.9 (0.7–1.1)
Wald χ^2^/p	4.43/0.03	NS	NS	NS	NS

Data from the Part 2 sample (n = 2,942).

* p = 0.04.

NS: non-significant.

† Based on multivariate logistic regression analysis controlling for gender, age, income, marital status, and education.

### Neighborhood social deprivation level

Roughly one-third of the SPMA residents (31.3%; SE:1.3) lived in neighborhoods at the higher levels of neighborhood social deprivation (NSD); corresponding estimates for no/low and low-medium/medium NSD were 33% (SE:1.4) and 35.8% (SE:1.7), respectively. Being an active SUD case was associated with residing in a higher NSD area; the odds of an SUD were roughly twice as large for higher NSD area residents as compared with residents living with no/low NSD levels, controlling for the respondent's socio-demographic variables, including income and education (Table 4). There was a modest association between disorder severity and living in medium or higher NSD level areas of the SPMA, with an OR of 1.3 (p = 0.04 in the contrast of medium NSD versus no/low NSD area residents, as shown in the last column of Table 4).

### Migration status

In our sample, 52% (SE:0.9) respondents had migrated into the SPMA after being born. Among the in-migrants, slightly more than one-third had come from rural areas (36.1%; SE:1.8); slightly more than one-third from small cities (37.0%; SE:1.8); about one in four had come from some other large city (27%; SE:1.7%). At the time of this survey, in-migrants tended to live in neighborhoods with some sort of deprivation: 36.8% (SE:1.8) in high/very high NSD and 36.6%; (SE:1.8) in low-medium/medium NSD. Only one fourth (26.6%, SE:1.9) was living in no/low NSD. Estimates of the association between the NSD level of SPMA residents and the odds of recently active mental disorders were generally unremarkable. The noteworthy exception involved recently active mood disorder: when compared to non-migrant SPMA residents, the in-migrants (born in other places) were less likely to present a mood disorder (p = 0.03; Table 4).

### Subgroup variation across urbanicity, neighborhood social deprivation, and migration status

In our search for male-female differences and subgroup variation across the urbanicity, NSD, and migration status variables, the product-terms suggested some leads for future research on mood disorders, anxiety disorder, and impulse control disorders (p<0.05). For example, among women, the estimated odds of being an active case of a mood disorder varied considerably in relation to migration status and in relation to our measure of urbanicity based upon where the resident was born and raised. The subgroup of women most likely to have active mood disorder was non-migrants who had been raised in one of the less urbanized parts of the SPMA. When compared with this higher prevalence subgroup of non-migrant women, the non-migrant women who had been raised in a more urbanized part of the SPMA had lower odds of mood disorder (OR = 0.38; 95%CI: 0.15–0.97; p = 0.038), as did the in-migrant women who had been raised in rural areas (OR = 0.3; 95%CI: 0.1–0.7; p = 0.004).

With respect to being an active case of anxiety disorder, in general there was a consistent two-fold excess odds for women versus men in all of the subgroups analyzed. For example, in the comparison of non-migrant women living in no/low NSD conditions versus non-migrant men living in no/low NSD conditions, the estimated OR was 2.3 (95%CI = 1.5–3.5; p = 0.0003). Nonetheless, there was an important exception among migrant men and women, where the migrant women living in no/low NSD conditions were much more likely than men to have active anxiety disorder as compared to migrant men living in the same conditions (OR = 5.7; 95%CI = 2.9-1.3; p<0.0001). This contrast lends some support to the idea that migrant men living in no/low NSD conditions may be in a relatively favorable situation with respect to being a case of anxiety disorder. As compared to these no/low NSD migrant men, the migrant men living in middle and higher NSD conditions were more likely to have an active anxiety disorder, with OR of 2.8 for the middle NSD versus no/low NSD contrast of these men (95%CI = 1.1–7.1; p = 0.03) and with an OR of 2.2 for the higher NSD versus no/low NSD contrast (95%CI = 1.1–4.7; p = 0.03).

With respect to ICD, in the primary study estimates there was no male-female difference (Table 2; p>0.05), no apparent effect of migration status (Table 4; p>0.05), and a non-robust gradient with no more than a modest association between living in middle-higher NSD conditions and being an active ICD case (Table 4, p>0.05). In the search for subgroup variation, we found some evidence that the subgroup of women born and raised in the SPMA might be more likely to be affected by active ICD. For example, among women living under conditions of high/very high levels of NSD, the non-migrant women were substantially more likely to have active ICD (OR = 2.8; 95%CI = 1.3–6.2; p = 0.007) than in-migrant women living higher NSD conditions of the megacity that might be in a relatively favorable situation. Migration status was not associated with ICD among women living in the other NSD conditions. Looking across NSD conditions, the only statistically robust association was found in the contrast of non-migrant women in the higher NSD conditions versus non-migrant women in the no/low NSD status, again with greater ICD prevalence among non-migrant women living in the higher NSD conditions (OR = 3.0; 95%CI = 1.1–8.1; p = 0.03).

### Service use by severity of disorders

Table 5 shows estimates for the proportion of SPMA residents receiving mental health services as well as evidence that severity of disorder was associated with greater likelihood of receiving services during the 12 months prior to the date of assessment. An estimated one in 11 SPMA residents (8.7%) had received treatment for mental health problems during that interval. About one in 12–14 residents received mental health care from a general practitioner or other provider in the general health care sector (7.7%). About one in 20 SPMA residents had been treated in the mental health care sector (5.3%), and about one in 50 had received mental health are from the non-health care sector and from CAM providers (2.0%). A small proportion received care from more than one sector (3.2%), and severity level of the disorder was quite strongly associated with treatment in more than one sector. For example, an estimated 32.8% of the ‘severe’ cases received mental health services in at least one sector, and an estimated seven percent of the ‘severe’ cases received treatment in more than one sector. The corresponding estimates for the ‘moderate’ cases were 12.6% and 1.8%, respectively. For ‘mild’ cases, these estimates were 3.6% and 0.4%.

**Table 5:** Association of 12-month DSM-IV/WMH-CIDI disorder severity and age cohorts with treatment type.

	Severity					Age, years							
	Severe	Moderate	Mild			18–34	35–49	50–64	65+			None Disorder	Any Disorder
Treatment	% (SE)	% (SE)	% (SE)	χ^2^_2_	p	% (SE)	% (SE)	% (SE)	% (SE)	χ^2^_3_	p	% (SE)	% (SE)
**Any Healthcare**	30.2 (2.5)	17.2 (2.4)	11.6 (2.1)	41.9	<.0001	16.8 (1.6)	23.5 (2.1)	23.7 (2.8)	18.9 (7.5)	8.4	.06	3.0 (0.3)	7.7 (0.4)
General Medical	12.0 (2.1)	7.7 (1.3)	5.9 (1.4)	6.62	.053	6.3 (1.2)	10.1 (1.4)	10.7 (2.1)	8.2 (3.9)	4.1	.27	1.1 (0.2)	3.2 (0.2)
Mental Health	23.2 (2.5)	12.3 (2.2)	6.4 (1.2)	47.12	<.0001	13.4 (1.7)	16.4 (1.6)	16.1 (2.6)	10.7 (5.6)	2.79	.44	2.0 (0.3)	5.3 (0.3)
**Non-Healthcare** a	9.5 (1.5)	4.6 (2.0)	1.5 (0.6)	32.67	<.0001	4.5 (1.3)	8.5 (1.4)	5.4 (1.2)	1.1 (1.1)	8.1	.06	0.7 (0.2)	2.0 (0.2)
**Any Treatment**	32.8 (2.4)	20.0 (2.7)	12.7 (2.1)	52.07	<.0001	18.8 (1.4)	27.2 (2.1)*	26.7 (3.0)*	18.9 (7.5)	12.06	.02	3.6 (0.3)	(0.4)

Data from the Part 1 sample (n = 5,037).

a Non-healthcare includes human services and complementary and alternative medicine.

* Significantly different from the prevalence in the 65+ sub-sample at .05 level.

This association between severity and treatment was statistically significant for treatment in the mental health specialty sector and in the non-healthcare setting, whereas in the general medical sector no association with severity occurred. Among respondents with no currently active WMH-CIDI disorder, an estimated 3.6% also received some sort of mental health treatment, possibly representing successful treatment of a previously active disorder or treatment of a mental disorder not covered in the WMH-CIDI assessment. Compared to respondents aged 65 years or more, the age cohorts of 35–49 yo and 50–64 yo presented significantly more use of service.

Women used more services compared to men (OR = 2.4; 95%CI = 1.7–3.4; p<0.0001). Neither income, marital status, education, nor NSD were related to the likelihood of mental health treatment (p>0.05; data not shown in a table).

## Discussion

This study provides the first empirical data on the prevalence of mental disorders and associated severity levels in the adult community population living in households within the Brazil's largest metropolitan area, which may serve as a model of what might be seen in other megacities of the LAC region specifically, and in the developing world generally. The results reveal that mental disorders are notably prevalent and the estimated 10% prevalence of ‘severe’ cases indicates that in this megacity there are more than one million adults with impairment levels indicating special need for mental health care. Comorbidity is quite a common phenomenon, with most of the morbidity concentrated in around 40% of the active cases that present two or more disorders. In addition, this study offers (1) evidence on the burden of mental health in a developing country where prior epidemiological data are scarce; (2) a comparison of the results with estimates from other WMH surveys, since the same methods were applied in this consortium initiative; and (3) an examination of the relationships between psychiatric morbidities and facets of urban life, such as exposure to violence, neighborhood social deprivation, and migration status.

Compared to corresponding prevalence estimates of WMH-CIDI-diagnosed DSM-IV mental disorders from the other 23 participating countries of the WMH Survey [31], [32], [33], [34], our estimate of 29.6% is larger than the corresponding value in the United States (26.2%) and about two times the estimates seen for the other upper-middle income participating countries [34]. Also, by comparison with results from the other countries, the SPMA seems to have the largest proportion of severely affected cases (10%), well above the US estimate (5.7%), the New Zealand (4.7%) [32], and those from the 14 countries reported elsewhere [31].

In our megacity, the anxiety disorders qualify as the most frequently observed condition [31] and major depression emerged as one of the most prevalent disorders, with higher estimated prevalence than has been seen elsewhere in other participant countries [35]. The estimate of SUD prevalence in São Paulo (3.6%) is higher than Colombia's and Mexico's, the other two LAC countries in the WMH surveys, which reported estimates of 2.8% and 2.5%, respectively [31], [36]. With respect to impulse-control disorders, the SPMHS' prevalence of intermittent explosive disorders exceeds the estimate of US (3.1% vs. 2.6%) and stands as the highest IED prevalence estimate among the WMH sites that assessed this disorder [37], [38].

The characteristics of our sample reflect the pattern of population growth of this megacity over the last decade: about one-half of the adult SPMA residents are in-migrants coming from other small cities and rural areas, most of them now living in suburban and peripheral deprived neighborhoods of the SPMA [6]. Concurrently, there has been a widespread scaling-up of urban violence [39], increasing the feeling of insecurity among people living in the megacity. As it happens, the SPMHS-estimated level of exposure to violence rivals to what has been experienced in armed conflict countries such as Lebanon [40].

In Brazilian health statistics of recent years, violence and injuries have been found to be one of the main sources of morbidity and mortality by external causes [41]. In our survey, crime-related events were found to be associated with all classes of mental morbidities and disorder severity, confirming previous reports that SUD, PTSD, and depression are frequent among individuals exposed to traumatic events [42]. SUD may increase the risk of violence victimization, over and above any purported effects of SUD on crime or violent behavior [42], [43].

A country's internal migration of workers due to economic reasons often has been described in relation to a ‘healthy migrant effect’ [44]. This favorable pattern was observed in the SPMA, with migrants less prone to present mood and ICD, as compared to non-migrants in the same area. Nevertheless, the potential effects of migration were observed unevenly in some subgroups of our sample, with several determinants possibly interacting with each other, e.g., being male or female, urban/rural origin, and neighborhood context, and some vulnerable subgroups were disclosed in our exploration of product-terms. For example, migration-related stressors in combination with high NSD might work to increase the likelihood of being an active case of anxiety disorder in men. In this context, our observed patterns of male-female variations with respect to migration status, urbanicity, and NSD deserve more detailed future analysis, possibly with probing into issues that will clarify the forces that brought the in-migrants to the megacity, clarifying the temporal sequence of events and processes at play during the causal pathways that lead toward increasing risk, severity, or non-persistence of mental disorders.

In these SPMHS estimates, previous exposure to an urban environment is associated with increased odds of presenting an ICD, and to a lesser extent, mood disorders and more severe disorders. These findings may be consistent with earlier reports that psychiatric disorders are more common among the inhabitants of urbanized areas [11], [12], [45]. A “breeder hypothesis” has been used to link the detrimental consequences of exposure to urbanicity to poor mental health status [11]. Other WMH sites that have surveyed urban areas, such as Colombia and European locations [11], [46], also found higher prevalence of mental disorders in more urbanized areas than less ones.

In contrast to this general pattern, non-migrant women raised in less urbanized areas of the SPMA seem to have been more vulnerable to mood disorders than women raised in more urbanized regions, or perhaps have more persisting mood disorder once it starts. Also, non-migrant women living in high NSD areas were also more likely to present an ICD than those from no/low NSD conditions. This might be due to the fact that in most peripheral deprived areas of the SPMA there is a predominance of woman-headed households with low education [6].Poverty among urban women may account for perpetuation of mechanisms of poor mental health [1].

The lack of male-female difference in ICD and drug dependence is in contrast with findings from other WMH countries [47], wherein for most externalizing disorders the estimates for men exceed those for women. Our data suggest a male-female convergence in externalizing disorders in the megacity, which might imply a growing burden of mental disorders in women [47], [48]. The findings of greater male-female differences in migrants from rural areas in mood disorders and migrants living in no/low NSD in anxiety disorders is consistent with previous reports that migration places women in a more vulnerable position in relation to men. How gender interact with other social contexts to shape health of migrant population is still an open matter [49].

With respect to age, most mental disorders, particularly the moderate/severe cases, were more common in early adulthood and midlife, suggesting impact on role-functioning during the important years of employment in the labor force [50], [51]. With respect to marital status, our finding that previously married residents were more likely to present an anxiety, mood, or ICD suggests the lack of social support of those divorced as one of complex pathways to mental disorders, as described by Kendler and colleagues in their research on depression in women [52]. The association linking loneliness and poor social relationships (including separation/divorce/widow status) with ill-health outcomes and mortality was recently clarified in a meta-analysis [53]. Changing marriage patterns, with increased social isolation, is considered both a predictor and a putative cause for poor mental health in urban areas [54], and of course, becoming separated or divorced may be a consequence of an active mental disorder as well.

### Use of services

As expected, disorder severity was found to be related to treatment seeking and receipt of services, which we surmise to be linked with the distress and impairment that accompany mental disorders [33], [36], [40], [55], [56]. Nonetheless, the majority of SPMA adults with active mental disorders did not receive treatment services. The finding of one third of those with serious disorders in the previous year receiving treatment is similar to findings in upper-middle income countries in the WMH survey consortium [34], but the SPMA estimate is not quite one-half the mean value observed in higher income countries.

Among severe/moderate cases, treatment in the specialty mental health sector was more common than general medical treatment, indicating an incipient mental service provision, contrasting with mental health care deregulation described in relation to our previous findings from a more circumscribed survey of neighborhoods in central São Paulo. Nevertheless, inequality and lack of integration also were observed within the SPMA [57], [58], [59]. Possibly, the gap in mental health treatment in other regions of the country is even worse.

### Limitations

Of course, this study has some limitations, and a few of the more salient ones should be mentioned. First, data are not representative of Brazil nor of the world's megacities in general; however, the detailed assessment of the population needs in this area is important for further tailoring policies and strategies to improve the mental health of the population to be served [60]. Second, the target population was restricted to people living in a large metropolitan area; generalization to rural or small city life is not warranted, even though an estimated 85% of the Brazilian population lives in urban areas [15]. To the extent that these two limitations exist, they are likely to increase the prevalence rates.

Third, the migrant group is heterogeneous, coming from diverse settings. Different ages at migration, socio-economic condition, and lengths of residence in the SPMA could interfere in the adaptation and acculturation process. Future analyses will be carried on using survival models to account for time-varying and time-invariant characteristics.

Fourth, only residents in households were surveyed, whereas the homeless and those institutionalized were not assessed. Fifth, household surveys relying on self-report assessments may induce unwillingness to participate and of non-disclosure; for instance, for alcohol or other drug use and problems. To the extent that these two biases exist, it will make our estimate conservative.

Sixth, this report does not include some clinically important disorders - notably, non-affective psychosis and dementia. Although the WMH-CIDI inquired about psychotic symptoms, this information does not allow the diagnosis of non-affective psychosis. Previous studies have shown that these symptoms are overestimated in lay-administrated interviews [61]–[64]. However, non-affective psychotic subjects might be captured as cases, as many are comorbid with anxiety, depression, and substance use disorders [64]. Therefore, if severity is underestimated in the WMH-CIDI results will be conservative. The exclusion of elderly with cognitive impairments that was unable to answer the questionnaire did not allow detecting dementia, what can have lowered the rate of cases in this age group.

Finally, the cross-sectional nature of our data does not allow determining the direction of association of sociodemographic variables with disorders assessed herein.

### Conclusion

This epidemiological survey of mental disorders experienced by adults living in a large and heterogeneous urban area has produced findings that may be a basis for current and future concern – not only in Brazil, but also in the LAC region, and perhaps in other megacities of the developing world. The observed estimates for the prevalence of mental disorders are among the largest ever seen in corresponding epidemiological surveys that have been conducted in other countries, with comparable field survey methods. A large proportion, one-third, of the active mental disorder cases qualify as ‘severe’ cases and most of these active and severe cases remain untreated. The heavy burden experienced by those with two or more disorders, as indicated by the association with severity, must be taken into account when planning services and prevention strategies.

These results call attention for the public health impact of mental disorders and offer an important foresight to stakeholders and health care providers [65]. If the world human agglomeration will be settled mostly in large urban centers and megacities during the rest of this new century, the case of SPMA deserves attention as a potential forewarning of what might be occurring elsewhere.

The low rate of treatment suggests that the incipient integration of mental health promotion and care into the rapidly expanding Brazilian primary health system [66]–[68] should be strengthened, reaching disadvantaged individuals without access to mental health services. Also, it is important to work with mental health promotion and early recognition of cases, particularly among young and males, those who are the group with less access to services in our survey.

Given the substantial burden of these mental health problems, it is important enhance the role of non-specialist health workers and other professionals, such as teacher and community leaders, in the recognition, detection and, eventually treatment of mental disorders. One potentially useful approach in poorly resourced countries is known as task-shifting or task-sharing [69]. Under this approach, there is up-regulation of capacities of primary medical care providers and non-medical professionals for effective treatment of mental disorders; core packages of mental health services are integrated into routine primary care. When accompanied by careful supervision and mentoring by mental health specialists, this approach can be used to scale up the mental health workforce in highly populated developing countries, particularly in the context of disadvantaged or especially vulnerable groups living in more deprived areas that otherwise might be outside the reach of mental health specialists [70]–[72].

## Supporting Information

Table S1 Center of Metropolitan Studies Neighborhood Social Deprivation (NSD) Index.
(DOCX)

Table S2 Estimated Twelve-Month Prevalence of DSM-IV/WMH-CIDI disorders by gender and age cohorts: results from the SPMHS.
(DOCX)
