# Supplementary material for: Mental Disorders in Megacities: Findings from the São Paulo Megacity Mental Health Survey, Brazil
Source: PLoS One. 2012 Feb 14;7(2):e31879. doi: 10.1371/journal.pone.0031879 (PMC3279422; doi:10.1371/journal.pone.0031879)
Supplement: Table S1 — Center of Metropolitan Studies Neighborhood Social Deprivation (NSD) Index. (DOCX) [file pone.0031879.s001.docx]

**Table S1:** Center of Metropolitan Studies* Neighborhood Social Deprivation (NSD) Index

| Group 1 (No NSD) | This group represents the best educational and income conditions, as well as a low presence of children and teenagers. It is strongly concentrated in the southwestern region of the SPMA. |
| --- | --- |
| Group 2 (Very low NSD) | Similar to Group 1 in terms of living conditions and age structure, but with a higher proportion of female head of households with lower education levels (up to 8 years). There is a strong concentration of this group around the core southwestern region of the SPMA. |
| Group 3 (Low NSD) | Represents families older than the two previous groups with the lowest concentration of children and teenagers. In terms of education, only 57.7% of the head of household had completed a primary education. |
| Group 6 (Medium-low NSD) | Represents old families, with the largest concentration of women heads of household with a primary education, a low prevalence of children, and the presence of adolescents similar to the average of the SPMA. In terms of income and education, has a profile similar to that of Group 4. |
| Group 4 (Medium NSD): | Medium socio-economic conditions and the presence of adult families. However, it has the largest concentration of children, the highest adolescent mortality rate, and income below the mean of the SPMA. Spatially, it is located in the most remote areas of the SPMA. |
| Group 5 (High NSD with young families) | The presence of young heads of household with low levels of income and education. This is the second group in the worst income indicators and education level, spatially located in the outskirts of SPMA. |
| Group 7 (High NSD and presence of adult families) | Heads of household (30% female) with low-income and education levels, a large concentration of children, and a high numbers of adolescents. This group is typically present in peripheral areas. |
| Group 8 (Very high NSD) | The largest concentration of children, great numbers of teenagers, and low mean age of the head of household (38 years). It has the worst level of education among all groups; less than 20% of heads of household have a full primary education. Among families in which the heads of household were women, 91.8% had not completed a primary education. This group also has the worst income indicators. |

*<http://www.centrodametropole.org.br/index.php?section=content&subsection_id=4&content_id=584>
