# Supplementary material for: Mental Disorders in Megacities: Findings from the São Paulo Megacity Mental Health Survey, Brazil
Source: PLoS One. 2012 Feb 14;7(2):e31879. doi: 10.1371/journal.pone.0031879 (PMC3279422; doi:10.1371/journal.pone.0031879)
Supplement: Table S2 — Estimated Twelve-Month Prevalence of DSM-IV/WMH-CIDI disorders by gender and age cohorts: results from the SPMHS. (DOCX) [file pone.0031879.s002.docx]

**Table S2**: Estimated Twelve-Month Prevalence of DSM-IV/WMH-CIDI disorders by gender and age cohorts: results from the SPMHS

|  |  |  | **Gender** | |  | **Age cohorts (years)** | | | |  |
| --- | --- | --- | --- | --- | --- | --- | --- | --- | --- | --- |
| **Disorder category** | **Disorder** | **n** | **Female** | **Male** | **χ^2^(p)** | **18-34** | **35-49** | **50-64** | **65 or more** | **χ^2^(p)** |
|  |  |  | **% (SE)** | **% (SE)** |  | **% (SE)** | **% (SE)** | **% (SE)** | **% (SE)** |  |
| **Anxiety disorders** | Panic disorder ^a^ | 61 | 1.6 (0.3) | 0.5 (0.1) | 14.3 (0.0009) | 0.8 (0.2) | 1.5 (0.4) | 1.5 (0.6) | -- |  |
|  | Generalized anxiety disorder^a^ | 134 | 3.0 (0.4) | 1.6 (0.3) | 7.2 (0.013) | 1.8 (0.3) | 3.1 (0.4) | 2.5 (0.5) | 1.9 (1.1) | 8.7 (0.054) |
|  | Social phobia^a^ | 186 | 4.9 (0.7) | 2.8 (0.3) | 6.7 (0.15) | 4.4 (0.6) | 4.0 (0.5) | 4.0 (0.6) | 1.2 (0.5) | 43.3 (<.0001) |
|  | Specific phobia^a^ | 572 | 14.7 (0.7) | 6.0 (0.7) | 82.7 (<.0001) | 8.9 (0.9) | 12.4 (1.0) | 14.1 (1.0) | 6.6(1.1) | 60.5 (<.0001) |
|  | Agoraphobia without panic^a^ | 88 | 2.3 (0.4) | 0.8 (0.4) | 6.5 (0.017) | 1.5 (0.5) | 1.7 (0.4) | 2.0 (0.5) | 0.8 (0.6) | 5.33 (0.18) |
|  | Post-traumatic stress disorder^b^ | 81 | 2.2 (0.3) | 0.8 (0.3) | 6.1 (0.02) | 1.4 (0.2) | 2.0 (0.4) | 1.7 (0.6) | 0.3 (0.2) | 29.67 (0.0002) |
|  | Obsessive-compulsive disorder^b^ | 155 | 4.2 (0.5) | 3.5 (0.5) | 1.0 (0.31) | 4.9 (0.8) | 3.1 (0.5) | 4.2 (0.8) | 0.9 (0.3) | 48.67 (<.0001) |
|  | Adult separation anxiety disorder^a^ | 111 | 2.3 (0.3) | 1.7 (0.4) | 2.6 (0.12) | 2.3 (0.4) | 1.8 (0.3) | 2.8 (0.7) | 0.05 (0.05) | 39.23 (<.0001) |
|  | **Any anxiety disorder^b^** | 841 | 26.1 (1.4) | 13.0 (0.1) | 38.7 <.0001 | 19.2 (1.5) | 20.3 (1.3) | 26.0 (3.5) | 11.0 (2.3) | 19.21 (0.0023) |
| **Mood Disorders** | Dysthymia^a^ | 62 | 1.9 (0.4) | 0.7 (0.3) | 7.6 (0.01) | 1.5 (0.3) | 1.5 (6.4) | 0.7 (0.3) | 1.3 (0.7) | 7.51 (0.08) |
|  | Major depressive disorder^a^ | 491 | 13.2 (1.0) | 5.3 (0.6) | 44.8 (<.0001) | 9.7 (0.6) | 10.9 (1.2) | 8.7 (1.0) | 3.8 (1.1) | 26.67 (0.0003) |
|  | Bipolar I and II disorders^a^ | 73 | 1.6 (0.2) | 1.3 (0.3) | 0.66 (0.42) | 1.6 (0.4) | 2.0 (0.3) | 0.8 (0.3) | 0.1 (0.06) | 44.49 (<.0001) |
|  | **Any mood disorder^a^** | 570 | 14.9 (1.0) | 6.6 (0.7) | 42.14(<.0001) | 11.3 (0.7) | 13.0 (1.3) | 9.6 (0.9) | 3.9 (1.1) | 47.55(<.0001) |
| **Impulse-control Disorders** | Oppositional-defiant disorder^a^ | 22 | 0.5 (0.2) | 0.5 (0.2) | 0.03 (0.85) | 0.5 (0.2) | 0.5 (0.2) | 0.8 (0.4)- | -- | -- |
|  | Conduct disorder^a^ | 19 | 0.2 (0.09) | 0.8 (0.2) | 8.5 (0.007) | 0.7 (0.2) | 0.4 (0.2) | 0.1 (0.1) | -- | -- |
|  | Attention deficit disorder^a^ | 49 | 0.6 (0.2) | 1.2 (0.3) | 3.4 (0.07) | 0.9 (0.3) | 0.9 (0.3) | 1.0 (0.4) | -- | -- |
|  | Intermittent explosive disorder^a^ | 138 | 3.1 (0.3) | 3.1 (0.5) | 0.0005 0.98 | 4.5 (0.7) | 2.8 (0.5) | 0.6 (0.2) | 1.3 (1.0) | 67.1 (<.0001) |
|  | **Any impulse-control disorder^a^** | 199 | 3.9 (0.5) | 4.5 (0.5) | 1.0 (0.32) | 5.9 (0.8) | 3.7 (0.7) | 1.9 (0.6) | 1.3 (1.0) | 29.51 (0.0002) |
| **Substance Use Disorders** | Alcohol abuse^a^ | 135 | 1.1 (0.3) | 4.6 (0.6) | 30.4 (<.0001) | 3.5 (0.6) | 3.2 (0.7) | 1.0 (0.3) | 0.3 (0.2) | 44.93 (<.0001) |
|  | Alcohol dependence^a^ | 64 | 0.5 (0.1) | 2.3 (0.3) | 19.8 (0.0002) | 1.5 (0.3) | 1.5 (0.4) | 1.3 (0.5) | 0.3 (0.2) | 17.51 (0.0036) |
|  | Drug abuse^a^ | 31 | 0.25 (0.09) | 1.0 (0.3) | 7.9 (0.009) | 1.1 (0.3) | 0.3 (0.1) | 0.1 (0.1) | -- | -- |
|  | Drug dependence^a^ | 21 | 0.3 (0.1) | 0.7 (0.2) | 2.3 (0.14) | 0.8 (0.3) | 0.5 (0.2) | 0.1 (0.1) |  |  |
|  | **Any substance use disorder^a^** | 164 | 1.5 (0.3) | 5.9 (0.6) | 52.1 (<.0001) | 4.5 (0.7) | 3.8 (0.7) | 1.9 (0.6) | 0.3 (0.2) | 48.3 (0.0001) |
|  | Any 12-month Disorder^b^ | 1277 | 36.8 (1.6) | 21.5 (1.6) | 39.8 (<.0001) | 30.9 (1.7) | 30.0(1.8) | 32.0 (3.4) | 15.5 (2.8) | 31.3 (0.0001) |

Part 1 Total Sample Size = 5037, Part 2 Total Sample Size = 2942

^a^ Part1 sample, prevalence calculated using Part 1 weights ^b^ Part2 sample, prevalence calculated using Part 2 weights
